# Wheat straw: A natural remedy against different maladies

**DOI:** 10.1002/fsn3.2030

**Published:** 2021-02-27

**Authors:** Tabussam Tufail, Farhan Saeed, Muhammad Afzaal, Huma Bader Ul Ain, Syed Amir Gilani, Muzzamal Hussain, Faqir M. Anjum

**Affiliations:** ^1^ Faculty of allied health sciences University Institute of Diet and Nutritional sciences The University of Lahore Lahore Pakistan; ^2^ Department of Food Sciences Government College University Faisalabad Faisalabad Pakistan; ^3^ Riphah College of Rehabilitation & Allied Health Sciences Riphah International University Faisalabad Faisalabad Pakistan; ^4^ University of The Gambia Serrekunda Gambia

**Keywords:** bioethanol, biogas, cellulose, hemicellulose, lignin, lignocellulosic mass, wheat straw

## Abstract

In millennia, much attention has been paid toward agro‐industrial waste which consists of lignin and cellulosic biomass. In this perspective, biomass waste which consists of lignocellulosic mass is an inexpensive, renewable, abundant that provides a unique natural resource for large‐scale and cost‐effective bioenergy collection. In this current scenario, efforts are directed to briefly review the agro‐industrial lignocellulosic biomass as a broad spectrum of numerous functional ingredients, its utilization, and respective health benefits with special to wheat straw. Wheat straw is lignocellulosic mass owing to the presence of cellulose, hemicellulose, and lignin. Its microbial culture is the most important and well adjusted, for a variety of applications in the fermentation substrate, feed, food, medicine, industry, and agriculture in order to increase soil fertility. In industrial fermentation, wheat straw can be used as substrates for the production of a wide range of hydrolytic enzymes, drugs, metabolites, and other biofuels as a low‐cost substrate or a natural source. Conclusively, wheat straw is the best source to produce bioethanol, biogas, and biohydrogen in biorefineries because it is a renewable, widely distributed, and easily available with very low cost, and its consumption is protected and environment friendly. Wheat straw is a moiety which has health benefits including anti‐inflammatory, antimicrobial, anti‐artherogenic, anti‐allergenic, antioxidant, antithrombotic, etc.

## Graphical abstract

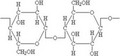

## 1 INTRODUCTION

In millennia, agro‐industrial waste captured greater interest owing to its abundant availability, pollution reduction ability, and lignocellulosic nature (Aboudi et al., 2016). The utilization of straw biomass in biobased composites is gaining momentum due to their cost efficiency, lightweight, low density, and less environmental impact during production (Sahai & Pardeshi, 2019). So far, the most commonly used material for biobased composite fabrication is wood (Chougan et al., 2020) but wheat straw as a renewable material has the potential to successfully replace wood in various applications. Agro‐industrial waste is the cheapest and largely generated lignocellulosic mass containing high contents of lignocellulose and starch. Lignocellulose is the major component of biomass and most abundantly renewable organic resource that contains about half of the plant matter and is produced by photosynthesis (Pala et al., 2014). It is important for the renewable energy, biofuels, and biochemical generation and is obtained from various sources, agricultural, and forestry waste stream (Davidi et al., 2016). The main industrial wastes include wheat straw, rice straw, corn straw, sugarcane, and sugarcane bagasse (Bharathiraja et al., 2017; Kumar & Sharma, 2017). Lignocellulosic mass has heterogeneous nature even if it is generated from single species of cereal straw (mainly wheat straw). It possesses lignin (5%–24%), cellulose (32%–47%), and hemicelluloses (19%–27%) (Sun & Cheng, 2002). Hemicelluloses and lignin are present in lesser amounts than cellulose but they provide protection to cellulose (Dang et al., 2009; Tsang et al., 2007). Among agricultural residues, straw is most abundant and the cheapest pollution mitigator. Straw is one of the most common lignocellulosic wastes that are produced by crops during their agricultural cultivation. 8–13 million tons of cereal straw is generated every year in Germany, whereas about 12.2 million tons of straw is produced in 2011 in UK from cereals and oilseed. Straw, when incorporated into the soil, has a nutritive value and contributes in organic matter content and soil quality. So it should not be wasted and must consume as animal feed/bedding. It contains lignin and holocellulose (cellulose and hemicelluloses) and some nonstructural components in small amounts (Hendriks & Zeeman, 2009; Silva et al., 2012). Average harvestable straw yields for wheat, barley, and oilseed rape in the UK are estimated to be 2.53 t/ha, 2.26 t/ha, and 1.65 t/ha, respectively (Wilson et al., 2013). Out of this value, wheat straw makes up 54% and about 50% of wheat straw is ground and is incorporated in soil after cutting and is not used for animal feed/bedding. Globally, wheat straw is most important by‐product of wheat processing produced in larger quantity (Hemdane et al., 2016; Reddy & Yang, 2005). About 529 million tons wheat straw is generated every year in all over the world (Govumoni et al., 2013), whereas 5–7 million tons of wheat straw are produced in the UK every year but currently just 1% is traded (Kang et al., 2014). Moreover, in all over the world and Europe, it is the amplest and the second most abundant and largest biomass feed stock after rice straw (Salvachua et al., 2011; Kim & Dale, 2004).

## 2 COMPOSITION

It is a lignocellulosic mass having cellulose (35%–40%), hemicelluloses (30%–35%), and lignin (10%–15%). As far as the nutritional composition is considered, wheat straw consists of high level of carbohydrates (lignin, cellulose, and hemicelluloses), proteins, minerals (calcium and phosphorus), silica, acid detergent fibers, and ash. It is also rich in bioactive compounds and vitamins. The macro‐ and micronutrient concentration depends upon the variety and cultivar, stage of plant growth, the nature of soil, fertilizer, and climatic situations (Safdar et al., 2009; Tufail et al., 2018; Yasin et al., 2010). The main phytosterols present in wheat straw wax are stigmasterol, campesterol, ß‐sitosterol, cholesterol, ergosterol, and stigmastanol. The structure of all these phytosterol resembles to ß‐sitosterol. Wheat straw is a complex structure possessing cell wall. The composition and properties of cell walls vary widely, containing cellulosic and noncellulosic components. The major components of the cell wall of wheat straw are arabinoxylan, ferulic acid, diferulic acid, lignin, and cellulose. These components of cell wall have varying degree of structural and functional complexity. It contains three main structural components: lignin (8%–15%), cellulose (35%–45%), and hemicelluloses (20%–30%). These components are bonded by noncovalent forces and covalent cross‐linkages (Perez et al., 2002).

### 2.1 Cellulose

Among structural components, cellulose is one of the main components and is considered as the most ample biomass in the world. These cellulose chains are bonded together by hydrogen bond to form microfibrils. These microfibrils vary in diameter (nanometers) and length (millimeters) and are structural unit of cell wall. These are bonded by a gel matrix composed of hemicelluloses, lignin, and other carbohydrate polymers to form a biocomposite (Moran et al., 2008; Thimm et al., 2000). Cellulose gives support and strength to these materials, to link lignin and hemicellulose to make microfibrils stable (Moran et al., 2008). The primary organization of cellulose is a linear unbranched polymer of ß‐glucose, connected with 1 → 4 β‐glycosidic bonds. Thus, the repeated unit in cellulose is a cellubiose residue rather than a glucose residue. Cellubiose performs a significant part in the hydrolysis of enzymes in regard of cellulose. It is the intermediate products of enzymatic hydrolysis in reference to cellulose which is then hydrolyzed to glucose. Cellulose is rich and distinctive biological compound globally, extracted from plants. They are widely utilized for making paper, ropes, sails, timber for housing, and for other utilizations. Wood is considered to be the most significant commercially utilized product worldwide (Eichhorn et al., 2010). The cellulose is most important extracted constituent for the manufacturing various materials such as hemp, cotton, jute flax, and sisal (Moran et al., 2008). The situation postulates that wood will become unavailable owing to low prices and lots of utilization globally. Instead of natural fibers, organic side products are major source of cellulose (Leitner et al., 2007).

Cellulose is substantial biomass of the world. The vital step in global carbon cycling and for bioenergy production is biodegradation of cellulose remains (Lynd, 2008). The use of cellulolytic microorganisms in rumen ecosystem is considered the most well‐organized process for cellulose transformation for the manufacturing of beneficial products. The complicated and dynamic hydrolytic methods are utilized for the processing of rumen cellulolytic microorganisms. For the manufacturing of highly valuable products, the potential biocatalyst is utilized for this purpose. The most active cellulolytic rumen bacteria are Fibrobacter succinogenes S85. Molecular as well as biochemical methodologies are being used for the investigation of enzymatic system, hemicellulases, and cellulases (Krause et al., 2003). Additionally, the comprehensive genomic arrangement of S85 of *F. succinogenes* is investigated to be hundred envisioned enzymes that are vigorous in contrast to polysaccharides derived from plants, inducing a maximum hypothetical activity of hydrolysis in that bacteria. F. succinogenes S85 have ability to metabolize sugars (Forano et al., 2008) although the rate of substrate metabolism is minimum in it than that of bacteria.

**Figure 1:**
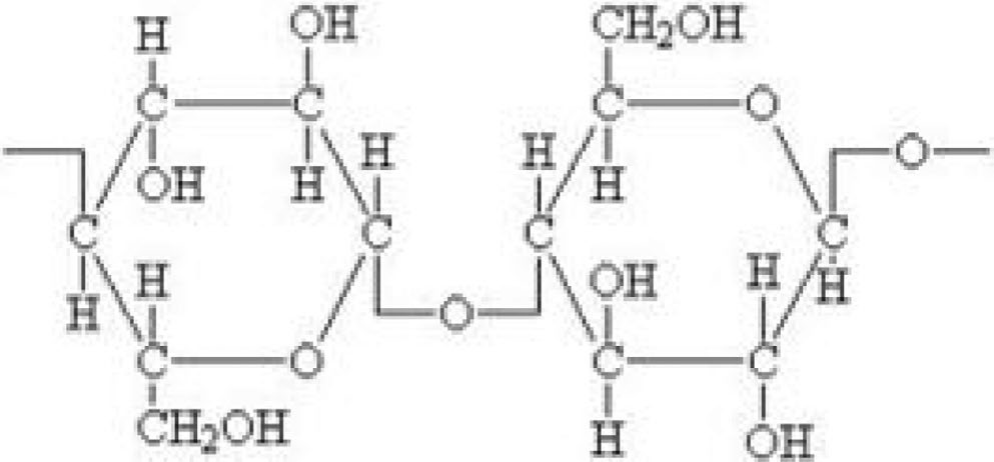
Structure of cellulose

Cellulose crystallinity causes a significant impact on the process of enzymatic hydrolysis that aids in formation of link between cellulose polymer chains by hydrogen bonding. The two different crystal types that bind cellulose sheet with each other are cellulose Iα and Iβ. The glucose products of both sheets could not be able to stack directly, but displacement occurs in the position of the chains in the cellulose sheets. The third layer has ability of restoration in the same direction similarly to the second, forming cellulose Iα, or in the opposed direction, forming cellulose Iβ. The two crystalline forms are thought to coexist in the cellulose. The foremost step in the pretreatment of lignocellulosic biomass is to interfere the crystallinity structure of cellulose that makes it more comprehensible in the enzymatic hydrolysis. The renewable cellulose resource around the world is wheat straw and is utilized in various industries. The valuable raw material for building board and in paper industry utilizes the cellulose fibers in combination with microfibers (Gousse et al., 2004; Liu et al., 2004; Puglia et al., 2003) (Figure 1).

### 2.2 Lignin

Lignin is the second most abundant, three dimensional, natural polymers and forms 10%–25% biomass of lignocelluloses (Rollin et al., 2011). Three different types of substituted phenols for instance sinapyl alcohols, p‐coumaryl, and coniferyl by the polymerization of enzymes are responsible for the formation of linkages and functional groups. Lignins that extracted from hydroxycinnamyl alcohols are generally known as syringyl, hydroxyphenyl, guaiacyl, and lignin (Mark & Kroschwitz, 2003). The complex phenylpropanic structure of lignin provides the lignocellulosic plant cell wall with the physical rigidity to stand upright. The class and the arbitrariness of the lignin linkage make it the most resistant biopolymer for degradation. This is the perfect epitome in reference to defend them contrary to herbivores and pathogens. Lignin is economically available in market in the form of cotton, wood pulp, jute, and hemp. Both the chemical and physical actions contradict in reference to unusual and fundamental basis and the methods of extraction (Watkins et al., 2015). The lignin extracted from wheat straw can be characterized and fractionated as lignin and carbohydrate complexes. Partition into two lignin and carbohydrate complicated element known as xylan as well as glucan lignin with glucan or arabinoxylan was done through ball milling derived from alkali medium and unprocessed wheat straw along with liquid nitrogen cooling reduce into widespread soluble solvent classification of dimethylsulfoxide‐aqueous tetrabutylammonium hydroxide by characterization through NMR spectroscopy methods and wet chemistry method (Kim et al., 2008).

Lignin is the second most abundant, three dimensional, natural polymers and forms 10%–25% biomass of lignocellulose. Three different types of substituted phenols for instance sinapyl alcohols, p‐coumaryl, and coniferyl by the polymerization of enzymes are responsible for the formation of linkages and functional groups. Lignins that are extracted from hydroxycinnamyl alcohols are generally known as syringyl, hydroxyphenyl, guaiacyl, and lignin (Mark & Kroschwitz, 2003). The complex phenylpropanic structure of lignin provides the lignocellulosic plant cell wall with the physical rigidity to stand upright. The class and the arbitrariness of the lignin linkage make it the most resistant biopolymer for degradation. This is the perfect epitome in reference to defend them contrary to herbivores and pathogens. Lignin is economically available in market in the form of cotton, wood pulp, jute, and hemp. Both the chemical and physical actions contradict in reference to unusual and fundamental basis and the methods of extraction (Watkins et al., 2015). The maximum amount of lignin inhibits enzymatic as well as microbial degradation.

**Figure 2:**
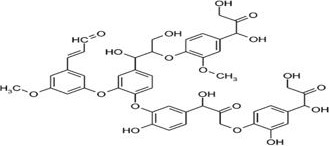
Structure of lignin

The lignin from wheat straw is extracted by the treatment of enzymes and ball milling. Various factors are involved in the yield of lignin such as grinding duration, cellulose hydrolysis time, and dioxane‐water composition of the extraction solvent. The yield of lignin during the process of isolation becomes excessive. Through the comprehensive analysis of NMR, the structure of lignin can easily be quantified (Zeng et al., 2013). The further investigation postulates the evidence for the lignin development in accordance with biofuels. 2D‐NMR, reductive cleavage, and analytical pyrolysis are utilized for the classification of lignin that are derived from wheat straw (Rio et al., 2012). The lignin extracted from wheat straw can be characterized and fractionated as lignin and carbohydrate complexes. Partition into two lignin and carbohydrate complicated element known as xylan as well as glucan lignin with glucan or arabinoxylan was done through ball milling derived from alkali medium and unprocessed wheat straw along with liquid nitrogen cooling reduce into wide spread soluble solvent classification of dimethylsulfoxide‐aqueous tetrabutylammonium hydroxide by characterization through NMR spectroscopy methods and wet chemistry method (Figure 2).

### 2.3 Hemicellulose

Hemicellulose is a complex polysaccharide that occurs in combination with cellulose extracted from the cell walls of lignocellulosic biomass. Representing dissimilarity to cellulose, hemicellulose consists of branched configuration that represents different structure of biomass of lignocellulose. Hemicellulose consists of four typical structurally different polysaccharide types: mixed‐linkage β‐glucans, xyloglucans, xylans, and mannoglycans (Ebringerova, 2005). Xylans and mannans are important hemicellulose in plant kingdom. Each type has a different linkage between its monomers. The hemicellulose, such as mannan and xylan, makes at least third of the total carbohydrate in most lignocellulose biomass. Thus, hemicellulose repossession causes a highly positive effect on the low‐cost production of ethanol through lignocellulose biomass. The observation of the hemicellulose hydrogen bonds to cellulose in lignocellulosic biomass suggests that they are cross‐linked. Single hemicellulose molecule exhibits the ability to link with more than one cellulose microfiber in order to form connection and also separation of nearby cellulose microfibers.

By the cross‐linkage of hemicelluloses, the cellulose microfibers can be separated from one another. They also have potential to influence the ability of the microfibers of celluloses to slip past one another. As a result of these cross‐linked bonds between hemicellulose and cellulose, lignocellulosic biomass in its initial structure is more unaffected to microbial confrontation, but pretreatment instigate extensive changes in the cell wall configuration that makes hemicelluloses and celluloses more manageable to hydrolysis of enzymes. Therefore, the hydrolysis of enzymes resulted from lignocellulosic biomass becomes maximum after processing. Hemicellulose is a diverged polymer having hexose and pentose sugars in its structural configuration. Hemicellulases or acids are utilized for the purpose of their hydrolysis and release its monomeric sugars. Arabinose as well as xylose is typical constituents of substantial fraction of lignocellulose quantity. The consumption is essential for manufacturing of bioethanol during processing (Aristidou & Penttila, 2000). Hemicellulose is a complex polysaccharide that occurs in combination with cellulose extracted from the cell walls of lignocellulosic biomass. Representing dissimilarity to cellulose, hemicellulose consists of branched configuration that represents different structure of biomass of lignocellulose. Hemicellulose consists of four typical structurally different polysaccharide types: mixed‐linkage β‐glucans, xyloglucans, xylans, and mannoglycans (Ebringerova, 2005). Xylans and mannans are important hemicellulose in plant kingdom. Each type has a different linkage between its monomers. The hemicellulose, such as mannan and xylan, makes at least third of the total carbohydrate in most lignocellulose biomass. Thus, hemicellulose repossession causes a highly positive effect on the low‐cost production of ethanol through lignocellulose biomass. The observation of the hemicellulose hydrogen bonds to cellulose in lignocellulosic biomass suggests that they are cross‐linked. Single hemicellulose molecule exhibits the ability to link with more than one cellulose microfiber in order to form connection and also separation of nearby cellulose microfibers (Tables 1 and 2).

**TABLE 1:** Lignocellulosic mass of wheat straw

Lignin (%)	Cellulose (%)	Hemicelluloses (%)	References
10.53	39.5	29.36	Shrivastava et al. (2014)
7.9±0.21	41.7±1.10	28.05±0.58	Thakur et al. (2013)
16.2	32.0	26.9	Rio et al. (2012)
11‐22.9	33.7‐40	21‐26	Yasin et al. (2010)
19.1	38.2	36.4	Zhang et al. (2010)
15‐Oct	35‐40	30‐35	Harper and lynch (1981)

**TABLE 2:** composition of fiber in untreated wheat straw

Straw	NCWM % w/w	Ash % w/w	Total lignin % w/w	Hemicellulose % w/w	Cellulose % w/w
1990	12.0	1.4	10.5	35.5	40.8
1993	18.8	1.4	8.9	32.8	38
2015	16.9	1.3	9.4	37.7	39.5

Abbreviation: NCWM, noncell wall material‐like pectin, proteins, etc.

**Figure 3:**
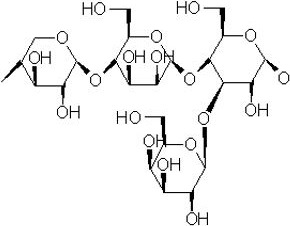
Structure of hemicellulose

By the cross‐linkage of hemicelluloses, the cellulose microfibers can be separated from one another. They also have potential to influence the ability of the microfibers of celluloses to slip past one another. As a result of these cross‐linked bonds between hemicellulose and cellulose, lignocellulosic biomass in its initial structure is more unaffected to microbial confrontation, but pretreatment instigate extensive changes in the cell wall configuration that makes hemicelluloses and celluloses more manageable to hydrolysis of enzymes. Therefore, the hydrolysis of enzymes resulted from lignocellulosic biomass becomes maximum after processing. Hemicellulose is a diverged polymer having hexose and pentose sugars in its structural configuration. Hemicellulases or acids are utilized for the purpose of their hydrolysis and release its monomeric sugars. Arabinose as well as xylose is typical constituents of substantial fraction of lignocellulose quantity. The consumption is essential for manufacturing of bioethanol during processing (Aristidou & Penttila, 2000; Koti et al., 2016) (Figure 3).

### 2.4 Phytosterol

**Figure 4:**
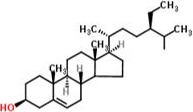
Structure of phytosterol

Phytosterols are natural constituents of cell membrane of plants. Their role in plants is similar to that of cholesterol in humans. They are present in vegetable oils, cereals, nuts, and vegetables. A number of products enriched in plant sterols/stanols, such as yogurts, milk, spreads, and margarines, can be found on the market, and their beneficial effects have been assessed in clinical studies. Wheat straw wax contains phytosterols (approximately 834–1206 mg/kg) mainly including stigmasterol, campesterol, ß‐sitosterol, cholesterol, ergosterol, and stigmastanol. The structure of all these phytosterol resembles to ß‐sitosterol. They are natural constituents of cell membrane of plants. Dunford and Edwards (2010) studies showed phytosterol content for cell wall of wheat straw was 60%–76%. The phytosterol's physical and chemical behavior is different with respect to the original source and extraction used. Greater the phytosterol content, greater is the grain and straw quality and vice versa. So the selection of best variety of wheat straw depends upon amount of phytosterol content it contained. Best variety is acknowledged to the high physterol containing variety. Phytosterol‐enriched foods and dietary supplements have been marketed for decades (Figure 4).

### 2.5 Policosanol

**Figure 5:**
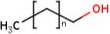
Structure of policosanol

Along with phytosterols, another bioactive compound present in wheat straw is policosanol (PC) which is the common name for a mixture of high molecular weight (20–36 carbon) aliphatic primary alcohols, which are constituents of plant epicuticular. Wheat is a good source of these compounds. The PC composition of extracts varied with the type of solvent and wheat fraction used. Ethanol and petroleum ether extracts of wheat straw have the highest octacosanol and hexacosanol contents, respectively. Wheat straw contains significant amount of PC (approximately 137–274 mg/kg). Octacosanol, tetracosanol, docosanol, hexacosanol, and triacontanol are the main PC components. Genotype and environment have a significant effect on PC content in wheat straw (Dunford & Edwards, 2010). Total PC content and compositions in the samples are determined by using a gas chromatography system (Dunford & Edwards, 2010). Recently, literature on the role of PC in prevention and treatment of cardiovascular disease was reviewed. Policosanol has been shown to decrease platelet aggregation, endothelial damage, and foam cell formation (Figure 5).

## 3 PRETREATMENT OF WHEAT STRAW PRIOR TO UTILIZATION

Pretreatment plays a considerable role in the utilization of wheat straw for various purposes. The aim of pretreatment is to increase the surface area and porosity of the substrate, reduce the crystallinity of cellulose, disrupt the heterogeneous structure of cellulosic materials, and improve the rate of production as well as the total yield of liberated sugars in hydrolysis step. A number of pretreatment methods have been developed and applied for wheat straw biomass. Combination of different pretreatment methods is used because single method cannot meet all objectives. The general effectiveness of the pretreatment process relies on a good low inhibitors formation and high substrate digestibility balance. The pretreatments are roughly classified into physical, physico‐chemical (liquid hot water, steam explosion, ammonia fiber explosion), chemical (acid hydrolysis, alkaline hydrolysis, wet oxidation, ozonolysis), and biological processes. The applied methods usually use combination of different principles, such as mechanical together with thermal and chemical effects in order to achieve high sugar release efficiencies, low toxicants production, and low energy consumption. The choice of appropriate pretreatment method for wheat straw depends upon several technological factors including energy balance, higher solid loading, and minimum use of chemicals as well as some environmental factors such as wastewater treatment, catalyst recovery, and solvent recycling. In terms of lower reaction time, higher solid loading and minimum use of chemicals, the most suitable method for pretreatment of wheat straw is steam explosion (Hendriks & Zeeman, 2009).

## 4 UTILIZATION OF WHEAT STRAW

Utilization of these wastes for meaningful purpose is beneficial as increase amount of these wastes creates health and environmental issues (Giuntini et al., 2006). For this purpose, first, the pretreatment is applied on the wheat straw structure to loose and break the bonding of lignocelluloses because this lignocellulosic cell wall network is resistant to enzymatic degradation (Barakat et al., 2013, 2015; Ji et al., 2016; Paes et al., 2017). Wheat straw is utilized for different purposes for example fuel for heating, animal feed, and bedding for domestic animals, although major portion of it is mixed in the soil and burned in arena. It has widely distributed, recyclable, dynamic, economic rates, productively, source of biogas, biohydrogen in biorefineries as well as bioethanol, to stimulate the biomass consumption overall in economically friendly atmosphere (Himmel & Bayer, 2009; Pasha et al., 2013; Rubin, 2008). The wheat straw signifies the major potential for the production of biofuel (Jorgensen et al., 2007; Lin & Tanaka, 2006; Lynd, 2008; Ragauskas, 2006). In addition, ethanol, a useful chemical constituent, is extracted from wheat straw (Demirbas, 2004; Tufail et al., 2020).

### 4.1 Production of biogas

The anaerobic digestion of organic wastes such as wheat straw represents a very interesting means of generating biogas while reducing the amount of waste to disposal. An enhancement in the hydrolysis limited digestion of straw can be achieved by optimizing operation and performing pretreatments (Ferreira et al., 2009).

### 4.2 Production of bioethanol

Being an important agriculture waste, wheat straw is considered as the most attractive, low cost, and potential feedstock for the manufacturing of bioethanol. 350 million tons of wheat straw produced annually at global level produces approximately 100 billion liters of bioethanol (Sarkar et al., 2012). However, production costs based on the current technology are still too high, preventing commercialization of the process. For the production of ethanol, enzymes, bacteria, and yeast are used. A sugar yield of 74%–99.6% is achieved after enzymatic hydrolysis and 65% to 99% of ethanol through yeast and bacteria. So far, the best results with respect to ethanol yield, final ethanol concentration, and productivity are obtained with the native nonadapted Saccharomyces cerevisiae. Some recombinant bacteria and yeasts have shown promising results and are being considered for commercial scale‐up. Wheat straw biorefinery could be the near‐term solution for clean, efficient, and economically feasible production of bioethanol as well as high value‐added products (Ain et al., 2019).

### 4.3 For animal feed

**Figure 6:**
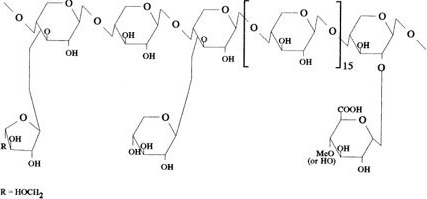
Chemical structure of hemicellulose extracted from wheat straw

**Figure 7:**
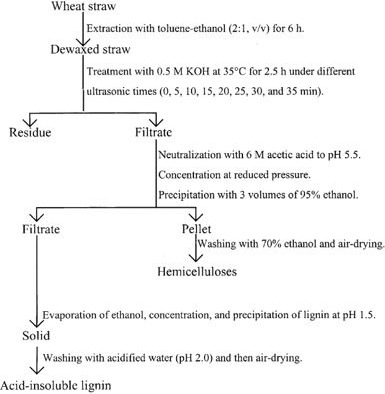
Schematic diagram of separation of lignocellulosic material

Each year approximately, 229.5 million ha of wheat is grown worldwide. After the grain is harvested, much of the straw is left in the field. Wheat straw may be used as an ingredient in cattle growing diets to help producers attain maximum utilization from their higher quality feedstuffs. Treatment with sodium hydroxide (NaOH) has been effective in increasing the digestibility of cereal grain straws.Reported an increase in digestible organic matter intake when cattle were fed 3.3% NaOH‐treated wheat straw rather than untreated straw. This increase in digestible organic matter intake should improve rate of gain. Lambs fed 4% NaOH‐treated wheat straw gained faster and more efficiently than those fed untreated wheat straw. While treated and untreated wheat straw has been compared in cattle digestion and intake trials and lamb growth trials, little research has been conducted to evaluate the inclusion of wheat straw in cattle growing diets (Figures 6 and 7).

## 5 HEALTH BENEFITS OF WHEAT STRAW

Wheat straw has enormous nutraceutical properties like anti‐allergenic, anti‐artherogenic, anti‐inflammatory, antimicrobial, antioxidant, antithrombotic, cardioprotective and vasodilatory effects, antiviral and anticancer owing to a marvelous source of bioactive compounds such as policosanols, phytosterols, phenolic compounds, and triterpenoids. These compounds are protecting against various diseases like hypercholesterolemia, intermittent claudication, benign prostatic hyperplasia, and cardiovascular diseases. Mechanism behind these effects includes antioxidant activity, mediation of hormones, enhancement of immune systems. Policosanol has been shown to decrease platelet aggregation, endothelial damage, and foam cell formation. PS is efficient in lowering low‐density lipoprotein‐cholesterol levels. Phenolic compounds having higher antioxidant activity are used to increase the shelf life of various food products. Triterpenoids demonstrate immense nutraceutical perspective as having antimicrobial, antiviral, anti‐inflammatory, and anticancer activities (Prachayasittikul et al., 2010). Currently, it is supposed that inhabitants suffer from androgen‐mediated diseases frequently such as prostate cancer, acne, hirsutism, benign prostatic hyperplasia (BPH), and androgenic alopecia. Wheat straw has been reported to relief from condition of biliousness (Drankham et al., 2003). It has been suggested that tooth disorders, that is, pyorrhea can be prevented and cured using wheat straw. Chewing of wheat grass not only benefits by exercising of teeth and gums but also assists in digestion. It acts as brilliant mouth wash especially for sore throat and pyorrhea as well as it keeps tooth from decay and toothaches. Moreover, it extracts out toxins from the gums and hence controls bacterial growth. With dermatological context, the ash of wheat straw has been reported to remove skin blemishes (Drankham et al., 2003).

## 6 CONCLUSION

Conclusively, agro‐industrial waste is the cheapest and largely generated lignocellulosic mass containing high contents of lignocellulose and starch. Cell wall of wheat straw is an excellent source of lignocelluloses, that is, lignin, cellulose, hemicelluloses. This lignocellulosic nature makes wheat straw and its cell wall more functional and more useful. Additionally, it makes the cell wall of wheat straw an important resource for the production of renewable energy, biofuel, bioethanol, and biochemicals. Moreover, some important bioactive moieties such as policosanol and phytosterol are present in cell wall of wheat straw. These functional ingredients make the cell wall of wheat straw much functional toward common diseases mainly cholesterol lowering and cardiovascular diseases. So, wheat straw should be used as a cheapest resource for biofuel, bioethanol, renewable energy owing to its abundant availability and lignocellulosic nature. As potential pollution reducing ability of wheat straw, it should be used to reduce the pollution by waste disposal. As a good source of policosanol and phytosterol, cell wall of wheat straw can be incorporated into food products to make them functional against many diseases because consumers are more conscious toward their diet and wish for natural remedies.
